# Reliability of the Maximal Step Length Test and Its Correlation with Motor Function in Chronic Stroke Survivors

**DOI:** 10.1155/2018/6985963

**Published:** 2018-12-20

**Authors:** Shamay S. M. Ng, Mimi M. Y. Tse, Patrick W. H. Kwong, Isaac C. K. Fong, Sun H. Chan, Thomson C. H. Cheung, Hoi-Ling Ko, David M. H. Yan, Cynthia Y. Y. Lai

**Affiliations:** ^1^Department of Rehabilitation Sciences, The Hong Kong Polytechnic University, Hong Kong; ^2^School of Nursing, The Hong Kong Polytechnic University, Hong Kong

## Abstract

**Objective:**

This study aimed to (1) investigate the interrater, intrarater, and test-retest reliabilities, as well as the minimal detectable change, of the Maximal Step Length test (MSL) in stroke survivors, (2) examine the concurrent validity of MSL with other stroke-specific impairment measurements in stroke survivors, and (3) compare the MSL performances of stroke survivors and those of age-matched healthy older adults in different directions.

**Design:**

Cross-sessional study.

**Setting:**

University-based research laboratory.

**Participants:**

Stroke survivors (n = 48) and age-matched healthy older adults (n = 39).

**Methods:**

Stroke survivors were assessed with MSL, lower limb muscle strength, Limits of Stability (LOS) Test, Berg Balance Scale (BBS), 5-meter walk test, and Activities-specific Balance Confidence (ABC) scale by two trained assessors in 1 session. Their performance on MSL was reassessed 1 week later to establish the test-retest reliability. Healthy older adults were assessed with MSL only. Intraclass correlation coefficient (ICC) was used to assess the reliability of MSL and Spearman's rho was used to quantify the strength of correlations between MSL and secondary outcomes. Between-group differences of MSL were assessed with the independent t-test.

**Results:**

The MSL exhibited excellent intrarater, interrater, and test-retest reliabilities [ICC: 0.885–1.000]. Significant correlations (*ρ*: 0.447–0.723) were demonstrated between MSLs in most directions and muscle strengths of the affected legs, BBS scores, and walking speeds. The step lengths differed significantly between stroke survivors and healthy older adults in the forward, backward, and sideways directions on both the affected and less affected sides.

**Conclusions:**

The MSL is a reliable, valid, and easily administered test of the stepping capabilities of stroke survivors. Stroke survivors had significant shorter MSLs in all directions than the age-matched healthy older adults.

## 1. Introduction

Stepping is an important reactive postural control response to perturbations and is essential to the maintenance of balance during daily activities. When one experiences a large perturbation wherein the center of gravity is displaced out of the base of support, stepping is the most effective strategy for maintaining balance [1]. In contrast, upon encountering a small-to-moderate perturbation, the body engages ankle or hip strategies to minimize unnecessary muscle activation while maintaining balance [2]. Stroke survivors commonly develop multiple sensory losses after stroke [3], which increase the latency associated with sensing postural changes during perturbations. Accordingly, these patients tend to more frequently implement stepping strategies to maintain postural stability and prevent falls [4]. Kajrolkar et al. [5] investigated the reaction to unexpected anterior slip during walking in a sample of ten stroke survivors. They reported that stroke survivors who lose balance initially learned to increase the compensatory step length of the affected leg by approximately 90% in the second trial and resulted in a 40% decrease in loss of balance [5]. Lakhani et al. [6] also revealed that, despite having a high BBS score (>50), four stroke survivors demonstrated a long delay in time to unload the stepping foot and were failed to step with their affected leg in response to perturbation [6]. Given the importance of stepping to a stroke survivor's ability to maintain balance, a reliable and valid outcome measure of stepping capabilities in this population is warranted.

The Maximal Step Length (MSL) test is a balance-specific measure used to assess stepping capability. This test was initially developed to assess balance and fall risks in older women [7]. In the original study, subjects were instructed to step out one leg “as far as possible” (i.e., maximally) in three directions (forward, sideways, and backward) while holding their stance leg stationary [7]. They were then required to return to the original position in one step without losing balance and to repeat the test with the other leg [7].

Studies of stroke survivors revealed that the MSL test had good to excellent test-retest reliabilities in the forward, sideways and backward directions [intraclass coefficient correlation (ICC): 0.939–0.990] [8, 9]. Previous studies also demonstrated a significant correlation of the MSL with the Five-Time-Sit-To-Stand completion time and walking speed (*r*: -0.83 to -0.88 and 0.69–0.72, respectively) in stroke survivors [9], and with lower limb muscle strength, clinical balance performance, and subjective balance confidence in community-dwelling older adults (*r*:0.60–0.84) [7, 10, 11].

These previous results suggest that the MSL test could be used to evaluate stepping capability during stroke rehabilitation. However, no study has compared the MSL test performance between stroke survivors and healthy older adults. Besides, no study has systematically investigated the intrarater, interrater, and test-retest reliabilities of this measure in the forward, sideways, and backward directions for both the affected and less affected legs in a cohort of stroke survivors. In addition, correlations of MSLs with other stroke-specific impairment measurements remain unidentified. Therefore, this study aimed to (1) investigate the interrater, intrarater and test-retest reliabilities and minimal detectable change (MDC) of the MSL test in stroke survivors; (2) examine the correlations of MSL results with other stroke-specific impairment measurements; and (3) compare the MSL performances of stroke survivors and those of age-matched healthy older adults in different directions.

## 2. Materials and Methods

### 2.1. Procedures

This cross-sectional study was conducted in a clinical research laboratory. The MSLs were the primary outcome and secondary outcomes including lower limb muscle strength, Limits of Stability Test, BBS, 5-meter walk test, and Activities-specific Balance Confidence scale. Stroke survivors were assessed by two raters, and a reassessment of MSLs was performed 7 days later to determine the interrater, intrarater, and test-retest reliabilities. Healthy older adults visited the laboratory once for demographic and MSL data collection. Subjects were asked whether they fell accidentally in the past 6 months. A fall was defined as “an unexpected event in which the participants come to rest on the ground, floor, or lower level.” [12]. This study protocol was approved by the ethics committee of the administrating institute. Prior to any testing, written consent was obtained from every participant and the study was conducted in accordance with the guidelines of the Declaration of Helsinki.

### 2.2. Participants

Stroke survivors were recruited from local self-help groups. Subjects were included if they (1) had a diagnosis of stroke confirmed by magnetic resonance imaging or computed tomography; (2) were aged ≥50 years; (3) were able to walk at least 9 m independently; and (4) had an Abbreviated Mental Test score ≥6. Age-matched community-dwelling healthy older adults were recruited from local community centers. Subjects were excluded if they had other comorbid neurological disorders such as Parkinson's disease or medical and/or orthopedic conditions that might impede assessment procedures. Since the test-retest reliability of the MSL was found to be good to excellent among stroke survivors [8, 9], we can assume that the ICC for assessing test-retest reliability in stroke survivors was 0.9. A sample size of 46 subjects achieves 80% power to detect an ICC of 0.9 with the null hypothesis of ICC equal to 0.8 and a significance level of 0.05. The sample size estimation was conducted with the PASS software (Version 14 NCSS, LLC. Kaysville, Utah, USA).

### 2.3. Maximal Step Length Test

The Maximal Step Length (MSL) is the maximal distance over which a subject can step and return to the original position in a single step without losing balance [7]. The MSLs measured using a tape measure (in cm). The subject was instructed to stand behind a line, step towards a particular direction (forward, sideways and backward) with each leg as far as possible and return to the starting position with their arms folded across their chests. All subjects were allowed two practice trials followed by six trials in each direction, for a total of 48 trials encompassing all directions and both legs. Each subject was given at least 30 seconds of rest between each trial to minimize the effects of fatigue. The order of testing directions was randomized by drawing lots. Research personnel stood close to the subjects and safeguard them. A trial is considered failed if the subject cannot maintain balance during or after making a step and required assistance from the research personnel to prevent them from falling. All failed trials were discarded and average step lengths of three trials for each direction were used for analysis.

### 2.4. Limit of Stability Test

A SMART Balance Master® system (Natus Medical Incorporated, San Carlos, USA) was used to quantify the maximum center of gravity excursion of a subject. The subject was required to stand on a dual-force plate that detected the center of gravity. The subject was then required to shift their center of gravity as rapidly as possible towards four cardinal directions and four diagonal directions as shown on the screen, while maintaining their balance at those positions without moving their feet. All subjects wore a harness to ensure safety. The maximum excursion (MXE; in percentages of distance towards the target) towards the aforementioned directions was then analyzed. Detailed procedures for the Limits of Stability (LOS) Test were documented in a previous study of stroke survivors, which demonstrated high test-retest reliabilities (ICC:0.78–0.91) [13].

### 2.5. Lower Extremities Muscle Strength

Affected knee and ankle muscle strengths were assessed since they are strongly correlated with functional mobility in stroke survivors [14, 15]. The strengths were measured using Lafayette Manual Muscle Test System™ (Model 01163, Lafayette Instrument Company, Lafayette, Indiana, USA). Each subject was placed in a high seated position with their back supported and knees at 90° flexion and was required to work maximally against the dynamometer for 3 seconds. Positions of dynamometer placements were described in a previous study [16]. Each muscle group was tested three times in an alternating sequence, with a 1-minute rest between trials. This handheld dynamometer measurement was found to exhibit high test-retest reliability (ICC:0.87–0.98) in stroke survivors [17]. The average strength of 3 trials for each muscle group was used for analysis.

### 2.6. Berg Balance Scale

The Berg balance scale (BBS) is a 14-item quantitative assessment used to measure balance and fall risk [18]. Each item is scored on an ordinal scale, with the maximum item score of 4 indicating the best performance. The maximum score for the scale is 56. This test was found to exhibit good to excellent interrater, intrarater and test-retest reliabilities (ICC: 0.95–0.98) in stroke survivors [18].

### 2.7. 5-Meter Walk Test

The 5-meter walk test quantitatively measures walking speed. In this test, the subject was asked to walk at their comfortable and maximal speeds on a 5-meter pathway, allowing an additional 2 meters for acceleration and deceleration. The time to complete the 5-meter walkway was measured, and the walking speed was calculated (in m/s). Each subject was required to complete three trials, and the mean of all trials was used for the data analysis. This test exhibited excellent test-retest reliability (ICC = 0.86) among stroke survivors [19].

### 2.8. Activities-Specific Balance Confidence Scale, Chinese Version

The Activities-specific Balance Confidence Scale, Chinese version (ABC-C), the Chinese version of the ABC, is a 16-item self-reported assessment used to measure a subject's confidence in performing various ambulatory activities without losing balance and is rated using a percentage scale (0–100%) [20]. This scale was proven to yield high interrater and test-retest reliabilities (ICC: 0.85–0.99) in community-dwelling elderly adults [20].

### 2.9. Statistical Analysis

R language (Ver. 3.5.1, R Core Team, Vienna, Austria) was used for data analysis. Subjects' demographic data were summarized using descriptive statistics. The independent t-test and chi-square test were used to compare demographic variables and MSLs between stroke survivors and healthy older adults.

ICCs were calculated to measure the intrarater (ICC_3,1_), interrater (ICC_2,2_), and test-retest reliabilities (ICC_3,2_) of the MSL among stroke survivors. For intrarater and test-retest reliability, ICC model 3 was used because generalization was not intended. ICC model 2 was used for interrater reliability, as the results could be compared and generalized to the results of other raters [21]. The ICC of test-retest reliability was used to calculate the standard error of measurement (SEM) as follows: $SEM=SD*\sqrt{1-ICC}$. The MDC was calculated using the formula $SEM*1\text{.}96*\sqrt{2}$.

As the sample size in this study was relatively small, Spearman's correlation coefficients were used to analyze correlations between the MSL and other outcome measures. The false discovery rate control method [22], which provides less stringent control of Type I errors, was used to adjust* p*-values for multiple analyses of the correlations between the different directions of MSL and other outcome measures.

## 3. Results

A total of 48 stroke survivors (32 male, 16 female) and 39 healthy older adults (12 male, 27 female) were recruited. These groups had mean ages of 61.4 ± 6.6 and 64.2 ± 7.5 years, respectively (Table 1).

There were significant differences existed in the MSLs in all six directions between the two groups (Table 2). Notably, the MSL demonstrated good to excellent interrater, intrarater, and test-retest reliabilities (ICC: 0.885–1.000) for both the affected and less affected legs of stroke survivors (Tables 3, 4, and 5). The MDCs ranged from 9.2 to 12.5 cm in the respective directions (Table 4).

We also observed moderate to strong positive correlations [Spearman's rho (*ρ*): 0.380–0.706] of the MSL with the muscle strength of the affected leg, BBS score and walking speed (except in the sideways direction) (Tables 6(a) and 6(b)). However, no significant correlations of the MSL test with the MXE of the LOS (except some weak correlations in the forward exclusion) and the ABC score were demonstrated.

## 4. Discussion

Results of the current study proved that MSL is highly reliable in evaluating the stepping performance in chronic stroke survivors. Moreover, the MSLs strongly correlated with muscle strength of the less affected lower limb, walking speed, and BBS, which suggested that this test demonstrated satisfactory concurrent validity. Stroke survivors performed significantly worse than the age-matched healthy older adults, indicating that the test exhibited good discriminative validity.

The stroke survivors in this study had MSLs that were comparable to the subjects included in the study by Pardo and colleagues [8]. However, in comparison to the study by Kobayashi et al. [9], the stroke survivors in this study had significantly greater MSLs in all three directions for both their affected and less affected limbs. According to Kobayashi and colleagues [9], the mean MSLs in their patient cohort ranged from 19.1 to 26.1 cm in different directions, whereas those of stroke survivors in our study ranged from 57.6 to 78.6 cm. This considerable disparity could be explained by demographic differences between the two studies. In our study, stroke survivors had a mean age of 61.4 years and a mean Timed Up and Go Test completion time of 15.8 seconds, compared to 69.9 years and 36.1 seconds, respectively, in the study by Kobayashi et al. [9]. According to Podsiadlo et al. [23], 76% of frail elderly subjects who completed the Timed Up and Go Test in 10–19 seconds could safely and independently go outside, whereas 85% of those who completed the Timed Up and Go Test in >30 seconds required assistance. Therefore, the stroke survivors in our study were likely more physically capable and thus performed better in the MSL test.

Four previously published studies [7, 24–26] investigated the MSL in older adults, although three of these studies measured only forward MSL [24–26]. Only one of these studies reported results comparable to ours [7], whereas the others reported MSLs between 49.4 and 71.9 cm [24–26], or shorter than that of healthy older adults in this study. This discrepancy in MSLs could be explained by differences in ages, as the mean ages of the elderly participants in the previous study ranged from 74.8 to 82.0 years, or much older than subjects in our study (64.1 years). This finding suggests that age might affect the MSL. This possibility should be investigated in future studies.

In this study, significant differences in MSLs in all directions between stroke survivors and age-matched healthy older adults were demonstrated. This difference is likely attributable to stroke-related impairments. Regardless of direction, the MSL involves the shifting of body weight from one leg to another. A stroke survivor would experience impairments in multiple aspects, including voluntary movements and asymmetries in gait, balance, and weight shifting [27–29]. As a result, the stroke survivors tended not to place weight on the affected side and performed worse in the MSL test than the healthy older adults.

Consistent with the results of previous studies of stroke survivors [8, 9] the results of our study demonstrated the good to excellent reliability of MSLs in all directions among stroke survivors. The simplicity of MSL likely contributed to these highly consistent results. The measurements were taken within 7 days to reduce the possibility of drastic changes in subjects' performances, which might also have contributed to the excellent test-retest reliability.

Our review of the literature suggests that ours is the first study to investigate the MDCs of MSL tests in stroke survivors in all three directions and on both sides. The MDCs of MSL tests ranged from 9.2 to 12.5 cm (Table 4). Thus, MSL was sufficiently sensitive to changes and could be useful for clinicians and researchers.

Two studies investigated the relationship between the MSL performance and muscle strength in the lower limbs in healthy adults [10, 30], and both suggested a positive relationship between the MSL and muscle strengths. Schulz et al. [10] reported a regression model in which MSL performance could predict knee extension (R^2^:0.66–0.78) and hip extension (R^2^:0.41–0.60) speed, strength, and power. These correlations can be explained by the nature of the MSL test, which measures balance from a biomechanical perspective through two motor subtasks. First, the MSL reflects muscle strength via the propulsion force in the stepping leg. Second, it reflects strength via the stability of the stance leg. During the MSL test, all four tested muscle groups in the stance leg cocontract to provide stability. Improved strength in these muscles might contribute to longer single leg stance phases and, therefore, longer MSLs.

Other than the aforementioned significant correlations, dorsiflexors strength on both legs did not exhibit significant correlations with the MSLs in sideways direction but still exhibited trends towards significance. This finding might be attributable to the small sample size of this study, which was calculated based on the primary objective of reliability. Therefore, this sample size might not have had sufficient statistical power to demonstrate significance in some correlation pairs. Besides, dorsiflexors may mainly contribute to the stability of ankle in anteroposterior but not in mediolateral direction.

There were weak or no correlations between MXE of LOS and MSLs. The differences in the level of external support of LOS and MSL can possibly explain the insignificant correlations between these factors. Stroke survivors were well protected by harnesses during the LOS test, whereas no harness was available during the MSL test. The harness might have provided a stronger sense of safety, higher confidence and, hence, better performance during the former test. Mobility performance would improve in the presence of higher self-efficacy, as the self-perceived balance confidence was previously found to correlate with the performance of daily living activities [30]. These reasons likely explain the insignificant correlation between the MSL and LOS.

The strong correlation between the MSL and BBS can be attributed to similarities in components of the stepping actions used in both tests. The MSL involves double leg standing, single leg standing, stepping out, and return, while certain items in the BBS also measure these components. For instance, items 2, 12, and 14 require both double leg and single leg standing, as well as stepping [18]. Therefore, we expected that subjects who performed better on the MSL test would also obtain higher BBS scores.

The MSLs did not correlate significantly with the ABC scale. The ABC is a real world-based measurement, whereas the MSL test is a lab-based measurement of a single specific task. In other words, the differences in the nature of these tests likely explain the lack of correlation. This conjecture was further supported by similar results from previous studies that validated walking performance tests, wherein the ABC did not correlate with specific lab-based physical tasks [31, 32].

However, another study of community-dwelling older adults found a statistically significant correlation between the MSL and short version of the ABC scale [33]. This finding may be attributable to differences in subject characteristics between the two studies. The confidence in balance maintenance exhibited by the healthy older adults recruited by Schepens et al. [33] may have been predominantly determined by their physical abilities. In contrast, in stroke survivors, this type of confidence might be determined not merely by physical ability, but also by the psychological effects of stroke, leading to insignificant results.

## 5. Study Limitations

The sample size in this study was calculated based on the reliability assessment as the primary objective. However, the sample size was relatively small for a parametric analysis of correlations and between-group comparisons. In addition, all participants had relatively good functional mobility. Therefore, the results might not be generalizable to all stroke survivors. Future studies should include more stroke survivors with stroke impairments of different severities. Besides, the hip muscles strength was not assessed in the current study. The maximal lateral step length may demonstrate a stronger correlation with the strength of hip abductors which induce movement in the coronal plane. Further study should investigate the correlations between hip muscles strength and maximal step lengths in various directions.

Furthermore, the majority of the stroke survivors in this study were male, whereas the majority of healthy older adults were female. This might have caused a sex bias because step length and physical characteristics are subject to sex differences [34].

In addition, movement quality and step speed, which are crucial to balance maintenance upon perturbation, were not considered in this study [35]. In addition, only isometric strength was measured, and this insufficiently reflects functional movements such as stepping. For future studies, both eccentric and concentric muscle work and endurance should be considered.

## 6. Conclusion

The MSL test was found to be reliable and valid for evaluating the stepping capabilities of stroke survivors, as it yielded good to excellent test-retest, intrarater, and interrater reliabilities and exhibited moderate to strong correlations with the BBS score and walking speed. Stroke survivors had significant shorter MSLs in all directions than the age-matched healthy older adults.

## Figures and Tables

**Table 1:** Demographic characteristics of two groups.

Characteristics	Stroke survivors		Healthy older adults		p-value
	n	%	n	%	
Gender (Male/Female)	32/16	66.7/33.3	12/27	30.8/69.2	0.462
Hemi side (Left/Right)	24/24	50/50	/	/	N/A
Dominant side (Left/Right)	/	/	2/37	5.1/94.9	N/A
Cause of stroke (Ischemic/hemorrhagic)	32/16	66.7/33.3	/	/	N/A
Number of fall (0/1/2)	38/10/0	79.2/20.8	36/2/1	92.3/5.1/2.6	0.257
Mobility status	22/24/2	45.8/50.0/4.2	38/1/0	97.4/2.6/0	0.515
(Unaided/With stick/SBQ)					
Ankle-foot orthoses (No/Yes)	47/1	97.9/2.1	/	/	N/A

	Mean ± SD [range]				

Age	61.3 ± 6.6 [50-77]		64.2 ± 7.5 [50-82]		0.600
Height (cm)	164.4 ± 7.4 [143-179]		159.8 ± 9.2 [145-183.7]		0.371
Weight (kg)	67.6 ± 10.7 [50.5-96]		57.6 ± 10.8 [37.5-90]		0.149
Body Mass Index (Kg/m^2^)	24.8 ± 3.0 [19.1-32.49]		22.5 ± 3.6 [17.0-34.6]		0.238
Leg length	85.4 ± 5.5 [73.4-95.7]		82.7 ± 6.3 [71.2-97.5]		0.329
Affected knee flexor strength (kg)	37 ± 20 [2-100]		/		N/A
Affected knee extensor strength (kg)	91 ± 38 [13-181]		/		N/A
Affected ankle dorsiflexor strength (kg)	8 ± 6 [0-24]		/		N/A
Affected ankle plantarflexor strength (kg)	32 ± 19 [6-85]		/		N/A
LOS MXE Forward (%)	65.5 ± 18.5 [21-105]		/		N/A
LOS MXE Affected side (%)	69.0 ± 15.7 [27-105]		/		N/A
LOS MXE Less affected side (%)	80.23 ± 13.1 [48-105]		/		N/A
LOS MXE Backward (%)	47.2 ± 17.3 [26-107]		/		N/A
Walking speed	1.0 ± 0.3 [0.4-1.7]		/		N/A

	Median ± IQR [range]				

BBS	53 ± 4 [47-56]		/		N/A
ABC	77.2 ± 18.8 [34.4-100]		/		N/A

BBS, Berg Balance Scale; ABC, Activities-specific Balance Confidence Scale; SBQ, small base quadripod; N/A, not applicable.

**Table 2:** Comparison of mean MSLs between stroke survivors and healthy older adults in all directions.

		Stroke survivors	Healthy older adults	p
MSLs of affected or non-dominant limb (cm)	Forward	61.3 ± 15.5	82.1 ± 15.1	<0.001*∗*
	Sideways	72.0 ± 12.6	93.5 ± 17.4	<0.001*∗*
	Backward	57.6 ± 15.7	76.9 ± 15.7	<0.001*∗*
MSLs of less affected or dominant limb (cm)	Forward	64.7 ± 14.8	83.7 ± 15.2	<0.001*∗*
	Sideways	78.6 ± 14.2	94.7 ± 18.4	<0.001*∗*
	Backward	59.2 ± 16.3	77.2 ± 16.9	<0.001*∗*

*Note*. Values are mean ± SD.

*∗* indicates a difference significant at the p ≤ .05 level of confidence.

**Table 3:** Intrarater reliability of MSL test in stroke survivors.

		Rater A		Rater B	
		Day 1	Day 2	Day 1	Day 2
Affected side	Forward	0.919	0.941	0.919	0.950
		(0.880-.0949)	(0.913-0.963)	(0.880-0.949)	(0.926-0.969)
	Sideway	0.905	0.923	0.904	0.924
		(0.862-.0939)	(0.887-0.952)	(0.860-0.939)	(0.888-0.952)
	Backward	0.902	0.917	0.903	0.924
		(0.857-.0938)	(0878-0.947)	(0.858-0.938)	(0.889-0.952)
Less affected side	Forward	0.898	0.934	0.898	0.935
		(0.846-.0936)	(0.902-0.959)	(0.849-0.936)	(0.903-0.959)
	Sideway	0.907	0.956	0.911	0.934
		(0.864-.0941)	(0.935-0.973)	(0.870-0.943)	(0.903-0.959)
	Backward	0.885	0.933	0.888	0.930
		(0.828-.0928)	(0.900-0.958)	(0.832-0.930)	(0.895-0.956)

*Note*. All the listed values are ICC_3,1_ (95% confidence interval).

**Table 4:** Test-retest reliability and MDC of MSL test in stroke survivors.

		Rater A			Rater B		
		ICC	SEM	MDC (cm)	ICC	SEM	MDC (cm)
Affected side	Forward	0.956 (0.887-0.979)	3.3	9.2	0.955 (0.888-0.979)	3.3	9.2
	Sideway	0.909 (0.807-0.953)	4.0	11.0	0.909 (0.807-0.954)	4.0	11.0
	Backward	0.940 (0.879-0.959)	4.1	11.4	0.942 (0.883-0.970)	4.0	11.0
Less affected side	Forward	0.942 (0.877-0.970)	3.6	10.1	0.943 (0.881-0.971)	3.6	10.0
	Sideway	0.907 (0.705-0.960)	4.5	12.5	0.909 (0.734-0.960)	4.5	12.3
	Backward	0.937 (0.796-0.973)	4.2	11.7	0.939 (0.819-0.973)	4.1	11.4

*Note*. All the listed ICC values are ICC_3,2_ (95% confidence interval).

SEM, standard error of the mean.

**Table 5:** Interrater reliability of MSL test in stroke survivors.

		Day 1	Day 2
Affected side	Forward	1.000 (1.000-1.000)	1.000 (0.999-1.000)
	Sideway	0.999 (0.999-1.000)	1.000 (1.000-1.000)
	Backward	1.000 (0.999-1.000)	0.995 (0.991-0.997)
Less affected side	Forward	1.000 (1.000-1.000)	1.000 (1.000-1.000)
	Sideway	1.000 (0.999-1.000)	0.999 (0.998-0.999)
	Backward	1.000 (1.000-1.000)	0.997 (0.995-0.998)

*Note*. All the listed values are ICC_2,2_ (95% confidence interval).

**Table:** (a) Correlation between MSLs of affected side and other outcome measures

		MSL Forward		MSL Sideway		MSL Backward	
		Spearman rho	*p*	Spearman rho	*p*	Spearman rho	*p*
Affected knee muscle strength (kg)	Flexor	0.561*∗*	<0.001	0.505*∗*	<0.001	0.515*∗*	<0.001
	Extensor	0.380*∗*	0.015	0.393*∗*	0.012	0.413*∗*	0.008
Affected ankle muscle strength (kg)	Dorsiflexor	0.549*∗*	<0.001	0.287	0.078	0.486*∗*	0.001
	Plantar Flexor	0.706*∗*	<0.001	0.481*∗*	0.002	0.633*∗*	<0.001
LOS MXE (%)	Forward	0.409*∗*	0.009	0.302	0.064	0.357*∗*	0.023
	Affected side	0.200	0.220	0.057	0.709	0.195	0.231
	Less affected side	0.253	0.124	0.189	0.246	0.239	0.147
	Backward	0.236	0.147	0.233	0.151	0.230	0.153
BBS		0.696*∗*	<0.001	0.536*∗*	<0.001	0.673*∗*	<0.001
5-meter walk test (m/s)	Maximal	0.577*∗*	<0.001	0.348*∗*	0.027	0.498*∗*	0.001
	Comfortable	0.499*∗*	0.001	0.285	0.079	0.412*∗*	0.009
ABC		0.069	0.660	0.069	0.660	0.096	0.563

LOS, Limits of Stability; MXE, maximal excursion; BBS, Berg Balance Scale; ABC, Activities-specific Balance Confidence Scale.

*∗* indicates a significant correlation at the p ≤ 0.05 level of confidence (p values were adjusted with False Discovery Rate correction).

**Table:** (b) Correlation between MSLs of less affected side and other outcome measures

		MSL Forward		MSL Sideway		MSL Backward	
		Spearman rho	*p*	Spearman rho	*p*	Spearman rho	*p*
Affected knee muscle strength (kg)	Flexor	0.548*∗*	<0.001	0.541*∗*	0.001	0.580*∗*	<0.001
	Extensor	0.411*∗*	0.009	0.477*∗*	0.002	0.363*∗*	0.021
Affected ankle muscle strength (kg)	Dorsiflexor	0.423*∗*	0.007	0.264	0.108	0.430*∗*	0.006
	Plantar Flexor	0.596*∗*	<0.001	0.384*∗*	0.014	0.619*∗*	<0.001
LOS MXE (%)	Forward	0.360*∗*	0.022	0.263	0.108	0.372*∗*	0.018
	Affected side	0.085	0.610	0.005	0.971	0.212	0.193
	Less affected side	0.239	0.147	0.134	0.420	0.288	0.078
	Backward	0.234	0.153	0.137	0.418	0.236	0.147
BBS		0.618*∗*	<0.001	0.523*∗*	<0.001	0.700*∗*	<0.001
5-meter walk test (m/s)	Maximal	0.540*∗*	<0.001	0.245	0.138	0.537*∗*	<0.001
	Comfortable	0.468*∗*	<0.001	0.169	0.300	0.455*∗*	<0.001
ABC		0.123	0.457	0.109	0.512	0.077	0.637

LOS, Limits of Stability; MXE, maximal excursion; BBS, Berg Balance Scale; ABC, Activities-specific Balance Confidence Scale.

*∗* indicates a significant correlation at the p ≤ 0.05 level of confidence (p values were adjusted with False Discovery Rate correction).

## Data Availability

The data used to support the findings of this study are available from the corresponding author upon request.
